# The *Escherichia coli* major exoribonuclease RNase II is a component of the RNA degradosome

**DOI:** 10.1042/BSR20140113

**Published:** 2014-12-23

**Authors:** Feng Lu, Aziz Taghbalout

**Affiliations:** *Department of Molecular Biology and Biophysics, University of Connecticut Health Center, 263 Farmington Avenue, Farmington, CT 06032, U.S.A.

**Keywords:** Exoribonucleases, PNPase, RhlB, RNA degradosome, RNA processing and degradation, RNaseE, RNase II, Co-IP, co-immunoprecipitation, Eno, enolase, HA, haemagglutinin, HHB, Hepes-hybridization buffer, IPTG, isopropyl β-D-thiogalactoside, LB, Luria-Bertani, PE, protein extract, PNPase, polynucleotide phosphorylase, RhlB, RNA helicase B, RNaseE, endoribonuclease E, RNaseE^C^, C-terminal half of RNaseE

## Abstract

Multiprotein complexes that carry out RNA degradation and processing functions are found in cells from all domains of life. In *Escherichia coli*, the RNA degradosome, a four-protein complex, is required for normal RNA degradation and processing. In addition to the degradosome complex, the cell contains other ribonucleases that also play important roles in RNA processing and/or degradation. Whether the other ribonucleases are associated with the degradosome or function independently is not known. In the present work, IP (immunoprecipitation) studies from cell extracts showed that the major hydrolytic exoribonuclease RNase II is associated with the known degradosome components RNaseE (endoribonuclease E), RhlB (RNA helicase B), PNPase (polynucleotide phosphorylase) and Eno (enolase). Further evidence for the RNase II-degradosome association came from the binding of RNase II to purified RNaseE in far western affinity blot experiments. Formation of the RNase II–degradosome complex required the degradosomal proteins RhlB and PNPase as well as a C-terminal domain of RNaseE that contains binding sites for the other degradosomal proteins. This shows that the RNase II is a component of the RNA degradosome complex, a previously unrecognized association that is likely to play a role in coupling and coordinating the multiple elements of the RNA degradation pathways.

## INTRODUCTION

RNA degradation plays an important role in controlling the synthesis of proteins by directly affecting the half-life of mRNAs. In *Escherichia coli*, normal degradation of mRNAs requires a four-protein complex, the RNA degradosome, whose components include the essential RNaseE (endoribonuclease E), RhlB (RNA helicase B), the exoribonuclease PNPase (polynucleotide phosphorylase) and the glycolytic enzyme Eno (enolase) [1,2]. RNaseE, the 1061 amino-acid product of the *rne* gene, is the core degradosomal component. The RNaseE^C^ (C-terminal half of RNaseE) includes a scaffold domain containing binding sites for all of the other elements of the complex, whereas the N-terminal half contains the catalytic endoribonuclease domain that is essential for the viability of the cell (reviewed in [3,4]).

During degradosome-mediated RNA degradation, RNaseE internally cleaves RNA substrates into fragments that are subsequently degraded to monoribonucleotides by the 3′→5′ exoribonuclease activity of PNPase. Structured substrates often also require melting of double-stranded regions by RhlB to provide the single-stranded substrates for PNPase and RNaseE. Thus, it makes sense that the components of this cooperative system exist within one structure. The direct role of Eno in degradosome function is not known (reviewed in [5]).

Because cleavage by RNaseE is confined to specific sites, the bulk of degradosome-dependent RNA degradation is thought to be mediated by PNPase, a phosphorolytic exoribonuclease. However, RNA degradation measured in *E. coli* cell extracts is predominantly hydrolytic [6] suggesting that hydrolytic exoribonucleases must play a significant role in cellular RNA turnover despite the fact that hydrolytic exoribonucleases have not previously been identified in isolated degradosome complexes [7–10].

RNase II, the 644 amino-acid product of the *rnb* gene, is the major hydrolytic exoribonuclease in *E. coli.* RNase II acts processively from the 3′ end of single-stranded RNA substrates and accounts for approximately 90% of the total exoribonucleolytic activity in cell extracts [6,11]. The primary role of RNase II appears to be the degradation of mRNA [12] although in the absence of other exoribonucleases, RNase II also functions in the processing of tRNA and other stable RNAs [13,14].

In the present work, we show that RNase II is associated with all of the known degradosome proteins. We also show that optimal association of RNase II with RNaseE requires the degradosomal protein RhlB as well as the RNaseE^C^ domain that contains binding sites for the other degradosome-associated proteins. The association of RNase II with RNaseE is not mediated by binding of the proteins to RNA substrates as shown by its insensitivity to RNase treatment.

The results indicate that the RNase II hydrolytic exoribonuclease is a component of the RNA degradosome, expanding the panoply of proteins within this organelle-like component that plays a central role in RNA processing and degradation in *E. coli* cells. The presence of RNase II and PNPase within the same RNA degradation complex is reminiscent of the eukaryotic exosome, which is typically composed of a phosphorolytic multiprotein core that is associated with other hydrolytic exoribonucleases (reviewed in [15–17]).

## EXPERIMENTAL

### Strains, plasmids and growth conditions

*E. coli* strains were grown in LB (Luria-Bertani) medium [18] to which 100 μg/ml ampicillin, 30 μg/ml chloramphenicol, 50 μg/ml kanamycin or 0.4% (w/v) glucose were added when indicated. Eno null mutants were grown in M9 media [19] supplemented with 0.2% (w/v) tryptone, 0.2% (v/v) glycerol, 1 mM MgSO_4_, 0.0001% thiamine and 40 mM succinate. Cell growth was monitored by measuring the optical density at 600 nm (OD_600_). Strains and plasmids are listed in Table 1. Gene knockouts were constructed by linear DNA recombination using λ Red-mediated gene replacement [20]. HA (haemagglutinin) and Flag-epitope tagging of chromosomally encoded proteins was done as previously described [21] and the associated antibiotic cassettes were eliminated, when indicated, by the use of the FLP-expressing plasmid pCP20 [22]. P1-mediated transduction was used to move chromosomal regions containing mutations or gene fusions encoding epitope-tagged proteins to different strains [19]. Plasmids were constructed as described in Supplementary material.

**Table 1:** Strains and plasmids

	Relevant genotype or description	Reference or source
Strains		
MC1000	*F- araD139 Δ(araABC-leu)7679 galU galK Δ(lac)X74 rpsL thi*	[41]
AT1*	*MC1000 ∆rne-cat*	[29]
AT8	*MC1000 rne**^1–417^**-cat*	[29]
AT25	*MC1000 rne-HA-cat*	[29]
AT33	*MC1000 rne::HA*	[29]
AT34	*MC1000 rne**^1–659^**::HA*	[29]
AT35	*MC1000 rne**^1–417^**::HA*	[29]
AT38	*MC1000 rne*::*HA* ∆*rhlB-cat*	[25]
AT50	*AT51* ∆*rhlB-cat*	This work
AT51	*MC1000 rne*::*HA* ∆*pnp*	[25]
AT52	*AT50* ∆*eno-kan*	[25]
AT53	*AT51* ∆*eno-kan*	[25]
AT68	*MC1000 rnb::Flag-kan*	[35]
AT99	*AT8 rnb::Flag-kan*	[35]
FL7	*AT68 rne::HA-cat*	This work
FL8	*AT68 rne**^1–659^**::HA*	This work
FL9	*AT68 rne**^1–417^**::HA*	This work
FL10	*AT50* ∆*eno*	This work
FL11	*FL10 rnb::Flag-kan*	This work
FL12	*AT38 rnb::Flag-kan*	This work
FL13	*AT51 rnb::Flag-kan*	This work
FL14	*AT50 rnb::Flag-kan*	This work
FL15	*FL17 ∆rhlB*	This work
FL16	*MC1000 rne*::*HA ∆pnp* ∆*eno*	This work
FL17	*MC1000 rnb::Flag rne::HA*	[35]
FL18	*FL16 rnb::Flag-kan*	This work
FL19	*FL17 ∆rhlB ∆eno-kan*	This work
FL20	*FL17 ∆eno-kan*	This work
**Plasmids**		
pMLB1113	*Low copy number plasmid vector*	[41]
pFL35	*P_lac_-rne**^418–1061^**::HA*	This work
pFL37	*P_lac_-rne**^633–1061^**::HA*	This work
pFL39	*P_lac_-rne**^820–1061^**::HA*	This work
pFL40	*P_lac_-rne**^762–1021^**::HA*	This work
pFL42	*P_lac_-rne**^634–1021^**::HA*	This work
pFL43	*P_lac_-rne**^696–1021^**::HA*	This work
pRNE32	*P_lac_-rne::HA*	This work

*Not viable in the absence of complementing plasmid encoding for RNaseE.

### Immunoprecipitation

Cells that coexpressed chromosomally encoded RNase II–Flag and plasmid-encoded RNaseE–HA derivatives were grown at 30°C to an OD_600_ of 0.8. Expression of the plasmid-encoded proteins was then induced by growth in the presence of 100 μM IPTG (isopropyl β-D-thiogalactoside) for 30 or 50 min. Cells that coexpressed chromosomally encoded RNase II–Flag and RNaseE–HA were grown under the same condition except that IPTG was omitted. Cell pellets were washed twice with cold PBS buffer (10 mM Na_2_HPO_4_, 2 mM KH_2_PO_4_, 137 mM NaCl and 2.7 mM KCl) and then frozen at −70°C. Protein extracts were prepared by ammonium sulphate precipitation of the 200000 ***g*** supernatants as previously described [7] except that the cells were broken in a French pressure cell; EDTA-free protease inhibitor cocktail tablets were used in all solutions (Roche Diagnostics); and the ammonium sulphate pellet was resuspended in IP buffer [25 mM Tris pH 7.5, 150 mM NaCl, 1 mM DTT (dithiothreitol), 1 mM EDTA, 5% (v/v) glycerol, 1% (v/v) nonidet P-40, and 1X protease inhibitor cocktail]. Under these conditions 60–65% of the RNase II present in the cleared cell lysates was recovered in the ammonium sulphate precipitates as shown by quantitative immunoblotting analysis [23]. Protein concentrations were determined using a BCA assay kit (Pierce).

IPs were carried out using anti-Flag M2 agarose beads (Sigma) or protein A/G agarose beads (Pierce) coupled to polyclonal anti-HA (Santa Cruz Biotechnology), polyclonal anti-Flag (Sigma) or rabbit IgG (Sigma) according to the manufacturers’ instructions. Beads were first incubated overnight at 4°C in the presence of 1 mg of PE (protein extract) in 0.5 ml of IP buffer, and then washed three times with 0.5 ml of IP buffer using spin columns (Pierce). Retained proteins were eluted by incubating the beads for 5 min at room temperature (≈25°C) in 50 μl of elution buffer containing primary amine (pH 2.5) (Pierce) or 0.1 M glycine (pH 3.5) and then collected by centrifugation in tubes containing 5 μl of 1 M Tris buffer (pH 9.5) to neutralize the pH.

When indicated, cell extracts were pre-incubated for 15 min at room temperature with 5 μg/ml of DNase-free bovine pancreatic RNase (Roche Diagnostics) prior to the overnight incubation of the PEs with the beads [24]. These conditions gave complete degradation of 200 μg/ml of yeast RNA (Sigma), as assayed by gel electrophoresis after incubation of PEs in IP buffer supplemented with yeast RNA.

### Western blots

Western blots were done as previously described by using the indicated primary antibodies and an alkaline phosphatase-conjugated anti-IgG secondary antibody (Sigma) [23]. Individual or pairwise combinations of polyclonal primary antibodies anti-HA (Santa Cruz Biotechnology), anti-Flag (Sigma), purified anti-PNPase, purified anti-RhlB and anti-Eno (kindly provided by S. Lin-Chao, Institute of Molecular Biology Academia Sinica) were used as primary antibodies. Anti-PNPase and anti-RhlB polyclonal antibodies (kindly provided by G. Dehò, Department of Biosciences, University of Milan and M. Cashel, Laboratory of Molecular Genetics, National Institutes of Health, Bethesda, respectively) were purified as previously described [25].

### Quantitative immunoblotting

To compare the cellular levels of epitope-tagged RNase II–Flag and RNaseE–HA derivatives in various strains, 80 and 160 μg of the total cell extracts were subjected to SDS/PAGE (10% gel) and then electroblotted onto nitrocellulose membranes. Similar expression levels of the plasmid-encoded RNaseE–HA derivatives were obtained by adjusting the incubation time of growing cells in the presence of IPTG. Anti-Flag or anti-HA primary antibodies and an alkaline phosphatase-conjugated anti-rabbit IgG secondary antibody were used to detect the RNase II–Flag and RNaseE–HA derivatives, respectively. Band intensities were proportional to amounts of protein applied to gels as estimated by densitometry using the ImageQuant program (Molecular Dynamics).

### Gel affinity (far Western) blotting

Purified RNase E–HA (7 μg), the bait protein, was boiled in 2% SDS buffer, electrophoresed on 10% SDS mini-polyacrylamide gels and then electroblotted to nitrocellulose membranes. To identify the RNaseE–HA band on the membrane, a parallel strip was cut out and stained with Ponceau stain [18]. To allow renaturation of the protein, the membranes were soaked in TEN50 buffer (10 mM Tris–HCl pH 8.0, 1 mM EDTA, 50 mM NaCl) at 4°C as previously described [26,27]. To block non-specific binding of proteins the membranes were then soaked for 1 h at room temperature in HHB (Hepes-hybridization buffer) containing 5% (w/v) non-fat dried skimmed milk [20 mM Hepes pH 7.7, 75 mM KCl, 0.1 mM EDTA, 2.5 mM MgCl_2_, 1 mM DTT, 0.05% (v/v) Triton X-100]. Membranes or separate strips of the same membrane were then incubated overnight at 4°C in HHB containing 1% non-fat dried skimmed milk, 1 mg of cell extract and 5 μg/ml DNase-free bovine pancreatic RNase (Roche Diagnostics) followed by three washes for 10 min at 4°C with HHB containing 1% non-fat dried skimmed milk. The membranes were then incubated for 1.5 h at room temperature with rabbit anti-Flag antibody in PBS buffer containing 0.02% (v/v) Tween 20 and 3% (w/v) non-fat dried skimmed milk, washed three times with the same buffer and then incubated for 1 h at room temperature with a horseradish peroxidase-conjugated anti-rabbit IgG secondary antibody (Sigma). Bands were detected by use of an ECL (enhanced chemiluminescence) kit (Thermo Scientific), using Biomax film (Thermo Scientific). Membrane strips used in parallel experiments were exposed on a same film. Band intensities were estimated by densitometry using the ImageQuant program (Molecular Dynamics). Band intensities were normalized to the band obtained with extracts from cells that expressed all of the degradosome proteins.

### Protein purification

To purify RNaseE–HA, AT1/pRNE32 cells that express RNaseE–HA under control of the *P_lac_* promoter were grown in LB at 37°C to an OD_600_ of 0.9–1. Expression of RNaseE–HA was then induced by growth for 75 min in the presence of 200 μM IPTG. Protein extracts were prepared as previously described [28] except that the ammonium sulphate pellet was resuspended in column buffer (50 mM NaPO_4_, 300 mM NaCl, 10% glycerol, 0.5% TritonX-100, pH 7.0) supplemented with a Protease Inhibitor Cocktail (Sigma). RNaseE–HA was purified on a TALON metal affinity column (Clontech) according to the manufacturer's instructions, taking advantage of a built in His-tag within RNaseE in which six histidines are clustered within the last 58 residues of the protein. This obviated the need to engineer in an exogenous His-tag.

## RESULTS

### RNase II interacts with RNaseE

**Figure 1:**
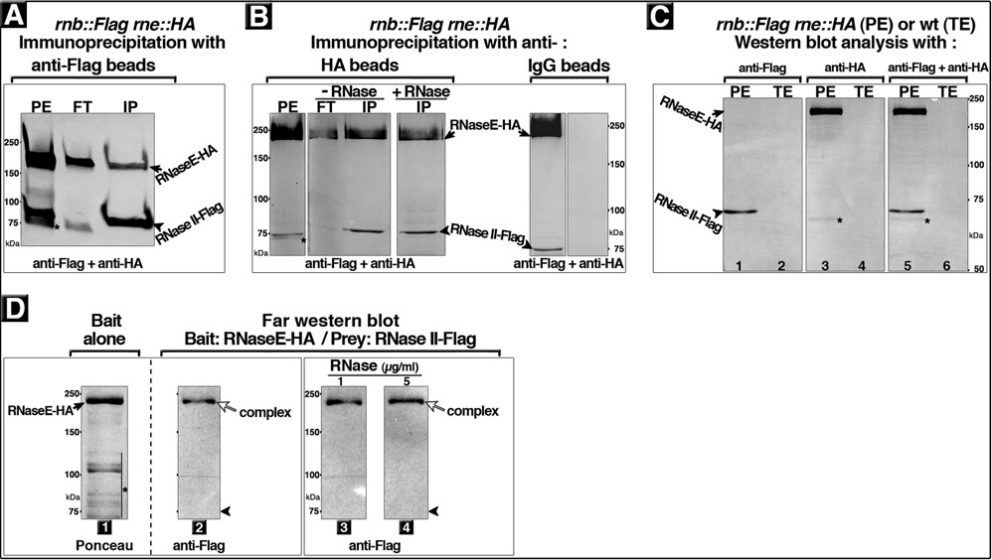
RNase II–RNaseE interaction (**A**, **B**) IP with anti-Flag (**A**), anti-HA (B left side) or IgG (B right side) beads of PE from cells that coexpressed under control of the native promoters chromosomally encoded RNase II–Flag and RNaseE–HA. In these cells, the wild-type *rnb* and *rne* genes were replaced with corresponding genes encoding RNase II–Flag and RNaseE–HA, respectively. PE and IP fractions, flow-through (FT) and bound (IP), obtained with the indicated anti-Flag, anti-HA or IgG beads, were subjected to Western blot using a mixture of anti-HA and anti-Flag polyclonal antibodies. When indicated PEs were pretreated with RNase from bovine pancreas (**B**) as described under the Experimental section. Lanes from same gel were rearranged as shown in (**B**). Arrows indicate positions of bands corresponding to RNaseE–HA; arrowheads indicate the positions of bands corresponding to RNase II–Flag; and star indicates bands corresponding to fragments that resulted from RNaseE–HA breakdown due to the sensitivity of RNaseE to proteolysis [7,42]. These RNaseE–HA breakdown bands were also detected when membranes were stained with anti-HA antibody alone (Figure 6). Protein size is shown in kDa. RNaseE runs slower than corresponding molecular weight on SDS–PAGE. (**C**) Western blot analysis with anti-Flag (lanes 1–2), anti-HA (lanes 3–4) or a mixture of anti-Flag and anti-HA polyclonal antibodies (lanes 5–6) of a total extract from wild-type cells (TE) and a PE of cells that expressed chromosomally encoded RNase II–Flag and RNaseE–HA. Proteins (150 μg) were separated on an SDS–PAGE (8% gel) and electroblotted onto a nitrocellulose membrane that was subsequently split and incubated with primary antibodies as indicated. Positions of bands corresponding to RNaseE–HA (arrow), RNase II–Flag (arrowhead) or a fragment that presumably resulted from RNaseE–HA proteolysis (star) are indicated. Protein size is shown in kDa. (**D**) Gel affinity (far Western) blot using purified RNaseE–HA as bait immobilized to nitrocellulose membrane and PE from cells that expressed chromosomally encoded RNase II–Flag as prey. The membranes were stained with Ponceau to detect the RNaseE–HA bait protein alone (lane 1) or with anti-Flag antibody to detect the RNase II–Flag prey protein (lanes 2–4) as described under the Experimental section. Lanes 1–4 are pieces of one membrane. The presence of RNase II–Flag band that colocalizes with the RNaseE–HA band indicates formation of RNase II–RNaseE complex(es). Arrows indicate positions of the Ponceau stained RNaseE–HA band (black) or the anti-Flag stained RNaseE–HA band that reflects the binding of RNase II–Flag to RNaseE–HA gel band (complex) (white); black arrowheads indicate the expected position of bands corresponding to free RNase II–Flag; and star indicates bands corresponding to fragments that resulted from RNaseE–HA breakdown. These RNaseE–HA breakdown bands were also detected when membranes were stained with anti-HA antibody (results not shown). Protein size is shown in kDa.

Evidence for interaction of RNase II with the major degradosome protein RNaseE came from Co-IP (co-immunoprecipitation) of epitope-tagged proteins RNaseE–HA and RNase II–Flag from cell extracts. RNaseE–HA and RNase II–Flag were functional in complementation assays as shown by their ability to support normal growth of *∆rne* or *∆*(*rnb pnp*) cells, respectively [23,29]. RNaseE–HA and RNase II–Flag were expressed under control of their native chromosomal promoters in cells whose wild-type *rnb* and *rne* genes were replaced by the corresponding gene encoding RNase II–Flag or RNaseE–HA, respectively. Immunoprecipitates were obtained by binding to protein A/G beads, or agarose beads coupled to anti-Flag, anti-HA or IgG polyclonal antibodies. The presence of differentially tagged proteins in the IP fractions was determined by Western blot using a mixture of anti-HA and anti-Flag polyclonal antibodies. This revealed that RNase II–Flag and RNaseE–HA were both present in the bound (IP) fractions obtained with anti-Flag beads (Figure 1A). Similarly RNase II–Flag and RNaseE–HA were both present in the IP fractions obtained in the reciprocal IP using anti-HA beads [Figure 1B left panel (-RNase)]. In contrast, no RNase II–Flag or RNaseE–HA bands were detected in the IP fractions obtained with IgG beads (Figure 1B right panel). The observed Co-IP of RNase II–Flag and RNaseE–HA indicates the presence of both proteins within the same complex.

Evidence that the proteins detected in IP fractions were not a result of non-specific binding to antibodies came from Western blot analyses of total extracts from wild-type cells (TE) with anti-Flag or/and anti-HA antibodies. This showed no detectable bands (Figure 1C lanes 2, 4, 6) in contrast to PEs from cells that expressed chromosomally encoded RNase II–Flag and RNaseE–HA, which showed the expected bands (Figure 1C lanes 1, 3, 5). This indicates that antibodies are highly specific in binding their corresponding epitope tagged-proteins and validates use of anti-Flag and anti-HA antibody mixtures for simultaneous staining in Western blot analyses.

It appears that RNase II is not associated with all cellular RNaseE, considering that part of RNaseE and none of RNase II were found in unbound fraction (FT). However, this may reflect an excess of RNaseE over RNase II in PE preparations that may result from uneven recovery of the proteins (see the Experimental section). Determination of the ratio of proteins in the obtained complex will require further quantitative studies.

Association of RNase II and RNaseE was also demonstrated in affinity blotting (far Western blotting) experiments using purified RNaseE–HA as bait. The RNaseE–HA was first electrophoresed on an SDS-gel and transferred to a nitrocellulose membrane. The membrane was then incubated with PE from cells that expressed the chromosomally encoded RNase II–Flag prey protein (see the Experimental section). Anti-Flag antibody was used to detect RNase II-Flag. This showed an RNase II-Flag band that colocalized with the Ponceau-stained RNaseE-HA band, indicating binding of the RNase II prey to the RNaseE gel band (Figure 1D lanes 1 and 2).

Evidence that the RNase II–RNaseE association was not mediated by an RNA bridge (i.e., substrate-mediated association) came from Co-IP and Western blotting experiments using cell extracts that had been treated with bovine pancreatic RNase. RNase II–Flag and RNaseE–HA bands were both present in the anti-HA IP fractions obtained with the RNase-treated extracts [Figure 1B (+RNase)]. Similarly, the RNase II–RNaseE interaction shown by far Western blotting was also unaffected by pretreatment of the PEs with bovine pancreatic RNase (Figure 1D lanes 3 and 4). The failure of RNase pretreatment to affect formation of the complex indicates that an RNA bridge was not responsible for the observed RNase II–RNaseE association.

### RNase II–RNaseE interaction requires the RNaseE^C^ scaffold domain

**Figure 2:**
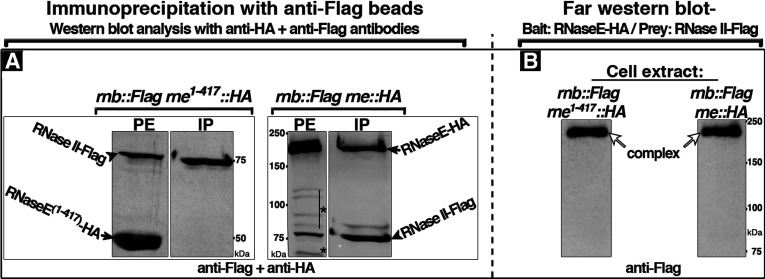
RNase II–RNaseE interaction requires the RNaseE^C^ scaffold domain (**A**) IP with anti-Flag beads of PEs from cells that coexpressed under control of the native promoters chromosomally encoded RNase II–Flag and RNaseE–HA or RNase II–Flag and RNaseE**^(1–417)^**–HA. In these cells, genes encoding the indicated epitope-tagged proteins replaced the wild type *rnb* and *rne* genes encoding RNase II and RNaseE, respectively. Protein extract (PE) and the obtained IP-bound fractions (IP) were subjected to Western blot using a mixture of anti-HA and anti-Flag polyclonal antibodies. Black arrows indicate positions of bands corresponding to RNaseE–HA or the RNaseE**^(1–417)^**–HA variant; arrowheads indicate positions of the RNase II–Flag bands; and stars indicate bands that correspond to RNaseE–HA breakdown fragments. Lanes from one gel were rearranged in panel (**A**). Protein size is shown in kDa. (**B**) Gel affinity (far Western) blot using purified RNaseE–HA as bait immobilized to nitrocellulose membrane and the indicated PEs from cells that coexpressed chromosomally encoded prey protein RNase II–Flag and RNaseE**^(1–417)^**—HA (left lane), or RNase II–Flag and RNaseE–HA (right lane). The membranes were stained with anti-Flag antibody to detect the band of RNase II-Flag prey protein as described under the Experimental section. The stained RNaseE–HA bands with anti-Flag antibody indicate formation of RNase II–RNaseE complex(es). The shown strips are from one membrane and were concurrently exposed to a film. White arrows indicate the position of band corresponding to RNase II–Flag: RNaseE–HA complex (es). Protein size is shown in kDa.

RNaseE is a core component of the RNA degradosome [27,30]. The RNaseE^C^ comprises a scaffold domain that includes binding sites for the other known degradosomal proteins, PNPase, RhlB and Eno and for other RNA modifying or binding proteins such as PAP I (poly(A) polymerase I) and Hfq [27,31,32]. This had suggested that the RNaseE^C^ domain acts as an interaction hub for proteins of the RNA degradation pathways. We therefore asked whether the observed RNase II–RNaseE interaction required the RNaseE^C^ domain by studying the truncated RNaseE**^(1–417)^** protein which lacks the C-terminal region of the protein and is defective in degradosome assembly [29]. Anti-Flag IP of PEs from cells that expressed chromosomally encoded RNaseE**^(1–417)^**–HA and RNase II–Flag showed a single band of RNase II–Flag and no band of RNaseE**^(1–417)^**–HA in the IP fractions (Figure 2A left panel). In contrast, RNaseE–HA and RNase II–Flag bands were both present in the IP fractions obtained in parallel experiments with PEs of cells that expressed full-length RNaseE–HA (Figure 2A right panel). The lack of Co-IP of RNase II with RNaseE**^(1–417)^**, which lacks the RNaseE^C^ domain, raised the possibility that the association of RNase II and RNaseE may be mediated by an interaction of RNase II with the RNaseE^C^ scaffold domain. Alternatively, the RNase II–RNaseE association might require other protein component(s) that might be absent in the *rne**^1–417^*** cell extracts.

Gel affinity (far Western) blotting experiments provided evidence that the failure of co- IP of RNase II and RNaseE**^(1–417)^** was not due to other changes associated with loss of RNaseE^C^ in the RNaseE**^1–417^** cells [1,29,33]. The ability of purified RNaseE–HA to interact with RNase II–Flag in far Western blots was tested using PEs of cells that coexpressed RNase II–Flag together with either truncated RNaseE**^(1–417)^** (Figure 2B left lane) or full-length RNaseE (Figure 2B right lane). This showed that RNase II–Flag from both cell extracts bound equally well to the immobilized purified RNaseE–HA bait protein (Figure 2B). Thus, the lack of Co-IP of RNase II and RNaseE**^(1–417)^** was not due to interference from secondary alteration of cellular composition associated with the loss RNaseE^C^ in the *rne**^1–417^*** mutant, but was due to lack of a putative RNase II-binding site within the RNaseE^C^ domain.

Taken together the data indicate that the C-terminal region of RNaseE is required for the observed interaction of the protein with RNase II. Whether the RNase II–RNaseE interaction is direct or is mediated by other degradosome proteins that bind to the RNaseE^C^ scaffold domain is addressed below.

### The RNase II–RNaseE complex includes the other degradosomal proteins

**Figure 3:**
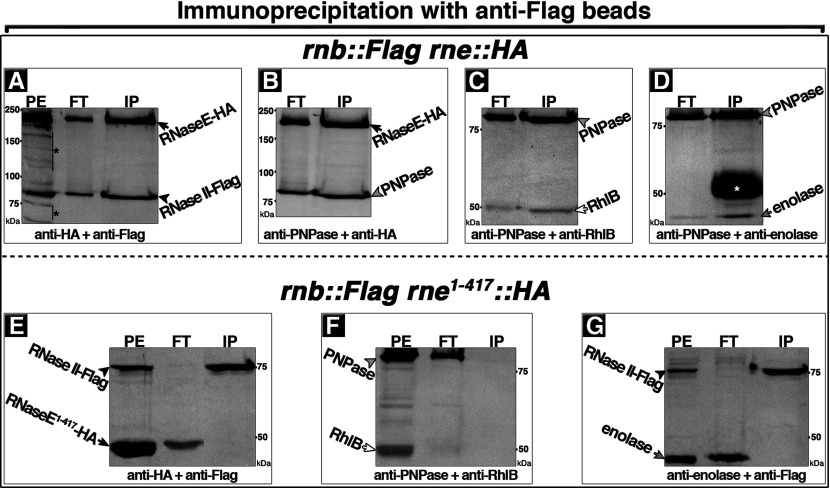
RNase II–degradosome association requires the RNaseE^C^ scaffold domain IP with anti-Flag beads of PEs from cells that coexpressed under control of the native promoters chromosomally encoded RNase II–Flag and RNaseE–HA (**A**–**D**) or RNase II–Flag and RNaseE**^(1–417)^**–HA (**E–G**). In these cells, genes encoding the indicated epitope-tagged proteins replaced the corresponding wild-type genes. RNaseE**^(1–417)^** fails to interact with RNase II and lacks the RNaseE^C^ domain containing the binding sites for the other degradosomal proteins (RhlB, Eno and PNPase). PE and the obtained IP fractions, flow-through (FT) and bound (IP), were subjected to Western blot using the indicated pairwise combination of polyclonal antibodies against HA- and Flag-tags or RhlB, PNPase and Eno. Arrows indicate positions of bands corresponding to RNaseE–HA or RNaseE**^(1–417)^**–HA variant (black), Eno (grey) or RhlB (white); arrowheads indicate positions of bands corresponding to RNase II–Flag (black) or PNPase (grey); stars indicate bands that correspond to RNaseE–HA breakdown fragments (black) or IgG heavy chains from anti-Flag protein A/G beads used to obtain the IP fractions in panel (**D**) (white). Protein size is shown in kDa.

To determine whether RNase II–Flag was interacting with the degradosome rather than with free cellular RNaseE, pairwise combinations of antibodies against the different degradosome proteins or their epitope tags were used in Western blots to probe the IP fractions obtained with anti-Flag beads. This showed that, in addition to RNaseE–HA, bands corresponding to PNPase, RhlB and Eno were also present in the anti-RNase II–Flag IP fractions (Figures 3A–3D). The Co-IP of all of the degradosomal components with RNase II strongly implies that RNase II was associated with the multiprotein degradosome complex.

Association of all the degradosomal proteins with RNase II required the RNaseE^C^ scaffold domain. The requirement for the scaffold domain was shown by the lack of Co-IP of degradosomal proteins in extracts from cells that coexpressed RNase II–Flag and RNaseE**^(1–417)^**-HA. RNaseE**^(1–417)^** lacks the RNaseE^C^ domain that contains binding sites for the other degradosomal proteins and does not support degradosome assembly. As shown in Figures 3E–3G, except for RNase II–Flag, none of the degradosomal protein bands were detected in the IP fractions obtained with anti-Flag beads. This confirms that the RNaseE^C^ domain that contains binding sites for the other degradosomal proteins is required for the association of RNase II with the degradosome complex.

### Role of individual degradosomal proteins in the RNase II–degradosome interaction

**Figure 4:**
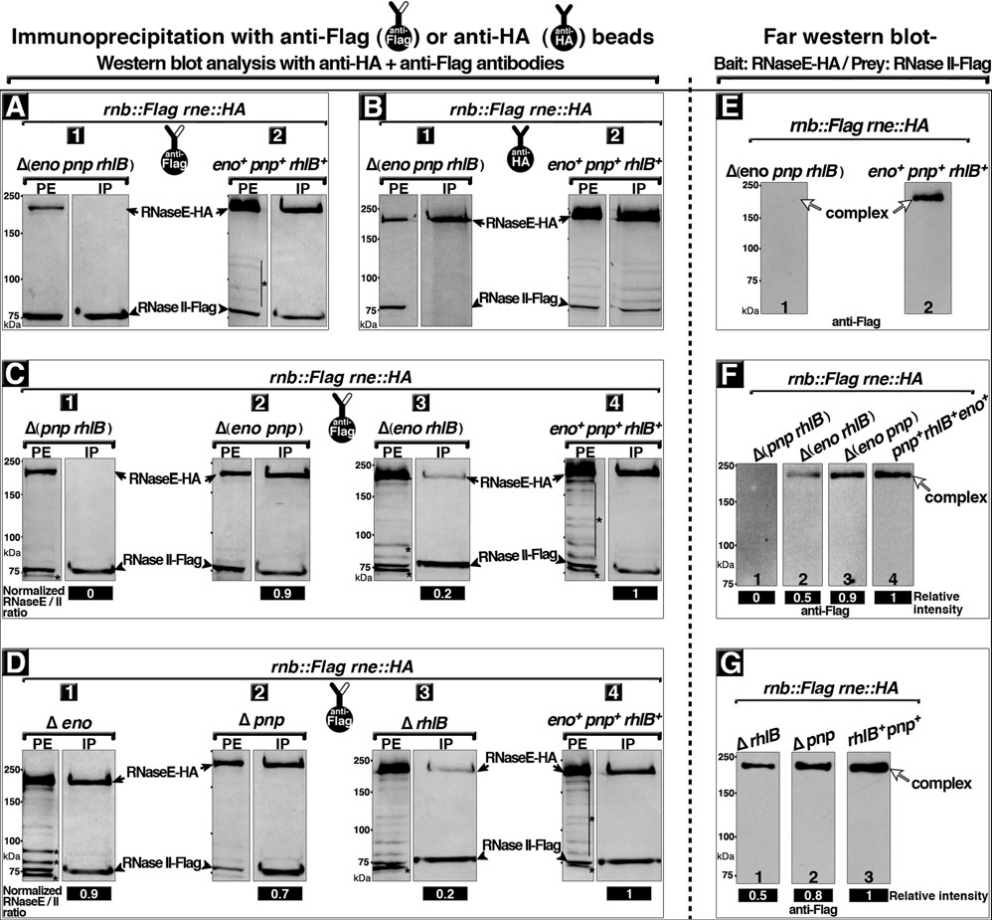
Role of individual degradosomal proteins in RNase II–degradosome association (**A–D**) IP with anti-Flag (**A**, **C**, **D**) or anti-HA (**B**) beads was performed on PEs from cells that coexpressed chromosomally encoded RNase II–Flag and RNaseE–HA under control of the native promoters. In these cells, genes encoding the indicated epitope-tagged proteins replaced the corresponding wild-type genes and when indicated genes encoding one or more of the other degradosomal proteins were deleted. FL11 cells [*rnb::Flag rne::HA ∆pnp ∆rhlB ∆eno*] (**A**, **B** panels 1); FL7 cells [*rnb::Flag rne::HA*] (**A**, **B** panels 2, **C**, **D** panels 4); FL14 cells [FL7 *∆pnp ∆rhlB*] (**C** panel 1); FL18 cells [FL7 *∆eno ∆pnp*] (**C** panel 2); and FL19 cells [FL7 *∆eno ∆rhlB*] (**C** panel 3); FL20 cells [FL7 *∆eno*] (**D** panel 1); FL13 cells [FL7 *∆pnp*] (**D** panel 2); FL12 cells [FL7 *∆rhlB*] (**D** panel 3). PE and bound fractions (IP) were subjected to Western blot analysis using a mixture of anti-HA and anti-Flag antibodies. Drawings depict anti-Flag or anti-HA beads used to obtain the fractions analysed in the corresponding panels. Black arrows indicate positions of bands corresponding to RNaseE–HA, arrowheads indicate positions of the RNase II–Flag bands and stars indicate bands that correspond to RNaseE–HA breakdown fragments. Relative ratio of RNaseE to RNase II bands of the same gel was normalized to a parallel IP fractions obtained from extract of cells that expressed all of the other degradosome proteins. Lanes of same gel are shown in panels **A**, **B**, (C-1 and C-4), C-2, C-3, D-1, D-2 and (D-3 and D-4). Protein size is shown in kDa. (**E–G**) Gel affinity (far Western) blots using purified RNaseE–HA as bait immobilized to nitrocellulose membrane and PEs from cells that expressed chromosomally encoded RNase II–Flag as prey and lacked one or more degradosomal proteins as indicated. Lane strips shown in each panel (**E**–**G**) are from the same respective membranes. The membranes were stained with anti-Flag antibody to detect the band of RNase II–Flag prey protein as described under the Experimental section. The stained RNaseE–HA bands with anti-Flag antibody indicate formation of RNase II–RNaseE complex(es). White arrows indicate the position of band corresponding to RNase II–Flag: RNaseE–HA complex(es). Protein size in kDa and relative band intensities normalized to the RNase II–Flag band obtained in extract of cells that expressed all of the other degradosomal proteins (panels **F**-4 and **G**-3) are shown.

Formation of the RNase II–degradosomal complex requires the RNaseE^C^ domain that contains binding sites for the other degradosomal proteins. This raised the question of whether assembly of the RNase II–degradosome complex requires the presence of the other degradosome proteins (RhlB, PNPase and/or Eno). We therefore performed RNase II/RNaseE Co-IP studies on extracts of cells that coexpressed RNaseE–HA and RNase II–Flag but failed to express all of the other degradosome proteins. The IP fractions were probed in Western blots with antisera containing a mixture of anti-Flag and anti-HA antibodies (Figure 4). The RNase II–RNaseE association was indicated by the presence of RNaseE–HA and RNase II–Flag bands in the immunoprecipitates. This showed that RNaseE–HA completely disappeared from the IP fractions obtained with anti-Flag beads from extracts that lacked all the other degradosome components (Figure 4A panel 1). As expected, in parallel experiments cells that expressed all the degradosome proteins showed both RNaseE–HA and RNase II–Flag in the Co-IP fraction (Figure 4A panel 2). Similar results were obtained in the reciprocal experiment in which anti-HA beads were used to immunoprecipitate RNaseE–HA (Figure 4B). These results showed that one or more of the other degradosome proteins is required for the observed RNase II–RNaseE association, and that the RNaseE^C^ domain, although required (Figure 2A), is not sufficient for RNase II–RNaseE complex formation.

To determine which of the other three degradosomal proteins is required for the RNase II–RNaseE association, we compared anti-Flag IP s of PEs from cells that expressed RNase II–Flag and RNaseE–HA but lacked one or more of the other degradosomal proteins. RNaseE was absent from IP fractions of cell extracts that lacked both RhlB and PNPase (Figure 4C panel 1) but was present in IP fractions of cell extracts that lacked Eno and contained RhlB and PNPase (Figure 4D panel 1), showing that the association of RNase II and RNaseE requires RhlB and/or PNPase but not Eno.

The Co-IP of RNase II and RNaseE was maintained at approximately normal levels in extracts from cells that lacked PNPase (Figure 4D panel 2) or PNPase and Eno (Figure 4C panel 2). This shows that RhlB alone can support the RNase II–RNaseE complex formation.

Small amounts of RNaseE–HA were also present in the anti-Flag IP fractions obtained from extracts of cells that lacked RhlB (Figure 4D panel 3) or RhlB and Eno (Figure 4C panel 3), as indicated by the ratio of RNaseE to RNase II relative to extracts that contained all of the other degradosome proteins (Figures 4C and 4D). This indicates that PNPase can support partially the RNase II–RNaseE association in the absence of RhlB.

Further evidence for the predominant roles of RhlB and PNPase in the formation of the RNase II–RNaseE complex came from far Western affinity-binding experiments using purified RNaseE–HA as bait and cell extracts containing RNase II–Flag and one or more of the other degradosome proteins as prey. The membranes were probed with anti-Flag antibody to detect association of RNase II–Flag with the RNaseE–HA band. As expected, when the cell extract contained all of the other degradosome proteins the affinity blot showed an RNase II–Flag band at the position of RNaseE–HA (Figure 4E lane 2), indicating formation of the RNase II–RNaseE (RNase II/E) complex. In contrast, when the cell extracts lacked both RhlB and PNPase RNase II–Flag failed to bind to the RNaseE–HA band (Figure 4E lane 1, 4F lane 1). These results indicate that binding of RNase II to RNaseE requires RhlB and/or PNPase. Consistent with this, extracts that lacked either RhlB or PNPase (Figure 4F lanes 2–3, 4G lanes 1–2) retained RNase II–RNaseE binding activity. The band intensity of RNase II-Flag, corresponding to the RNase II/E complex, was approximately 50% stronger in the absence of PNPase (Figure 4F lane 3, 4G lane 2) than in the absence of RhlB (Figure 4F lane 2, 4G lane 1). These results show that the RNase II–RNaseE interaction requires that the reaction mixture contain the RhlB or PNPase component of the degradosome, although RhlB is the more effective partner judging from the relative intensity of the bands. This requirement for RhlB and/or PNPase also indicates that formation of the RNase II–RNaseE complex in far Western affinity-binding experiments is not a result of an artefact of excess or renaturation of RNaseE. These results were consistent with the observation that Co-IP of RNase II and RNaseE required RhlB and/or PNPase (Figure 4C).

### RNaseE domain required for RNase II–degradosome interaction

Optimal formation of the RNase II–degradosome complex in Co-IP experiments required the RNaseE^C^ (Figure 2A). To further define the region of RNaseE that is involved we tested various HA-tagged RNaseE^C^ fragments expressed under *P_lac_* control for their ability to restore the RNase II–RNaseE association in cells expressing chromosomally encoded RNase II–Flag and RNaseE**^(1–417)^**. RNaseE**^(1–417)^** does not itself interact with RNase II (Figure 2A) but is required for viability of cells that lack full-length RNaseE. The IP fractions were analysed by Western blot using a single antibody against HA or Flag, or pairwise combinations of antibodies directed against the HA and Flag tags or against the different degradosome proteins.

**Figure 5:**
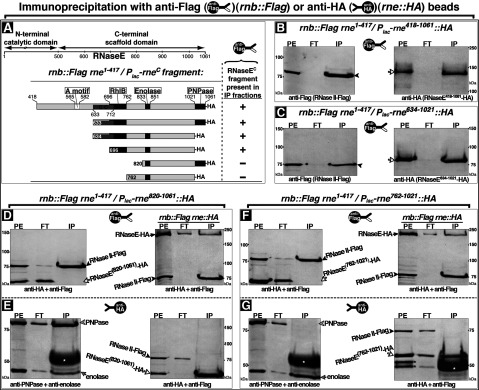
RNaseE domain required for RNase II–RNaseE interaction (**A**) Schematic representation of the RNaseE catalytic N-terminal domain and C-terminal (RNaseE^C^) scaffold domain containing binding sites for degradosomal proteins RhlB, Eno and PNPase (black) [27], the predicted coiled-coil (c c) domain (dark grey) [43] and the A motif membrane-anchor (white) [34]. Depicted are the plasmid-encoded HA-tagged RNaseE^C^ fragments tested in Co-IP experiments for their ability to restore RNase II–RNaseE association in cells that coexpressed chromosomally encoded RNase II–Flag and RNaseE**^(1–417)^**. RNaseE**^(1–417)^** failed to interact with RNase II in Co-IP experiments. (+) and (−), respectively indicate the presence or absence of the HA-tagged RNaseE^C^ fragments in the IP fractions obtained with anti-Flag beads. (**B–G**) IP with anti-Flag (**B–D**, **F**) or anti-HA (**E**, **G**) beads of PEs from cells that coexpressed under control of native promoters chromosomally encoded RNase II–Flag and the RNaseE**^(1–417)^**. The cells also expressed under the control of *P_lac_* promoter the indicated plasmid-encoded HA-tagged RNaseE^C^ segments. PE and the obtained IP fractions, flow-through (FT) and bound (IP), were subjected to Western blot using anti-Flag (**B**, **C** left panels) or anti-HA (**B**, **C** right panels) polyclonal antibodies or the indicated pairwise combinations of polyclonal antibodies against HA- and Flag-tags or PNPase, Eno degradosomal proteins. Drawings depict anti-Flag or anti-HA beads used to obtain the fractions analysed in the adjacent gels. Arrows indicate positions of bands corresponding to RNaseE–HA (black), RNaseE^C^–HA fragments (white) or Eno (grey); arrowheads indicate positions of the RNase II–Flag bands (black) or PNPase (grey); and stars indicate bands that correspond to breakdown fragments of RNaseE–HA (black) or IgG heavy chains that were present when protein A/G beads were used in panels (**E–G**) (white). Protein size is shown in kDa. The RNaseE^C^ fragments RNaseE**^633–1061^**–HA and RNaseE**^696–1021^**–HA (panel **A**) gave similar results as the RNaseE^C^ fragments shown in panels **B**, **C** (Supplementary Figure S1). The RNaseE^C^ fragments and RNaseE full-length migrate slower than corresponding molecular weight on SDS–PAGE.

RNaseE^C^–HA fragments that contained the RhlB-binding site were co-immunoprecipitated with RNase II-Flag even if the fragments were expressed separately from the N-terminal catalytic domain of RNaseE. This was shown by the presence of RNase II–Flag and the RNaseE^C^–HA fragment in the anti-Flag IP fractions (Figures 5A–5C, and Supplementary Figure S1). In contrast, fragments that lacked the RhlB-binding site were undetectable in the anti-Flag IP fractions, regardless of whether the PNPase-binding site was present (RNaseE**^(820–1061)^**–HA) (Figure 5D left panel) or absent (RNaseE**^(762–1021)^**-HA) (Figure 5F left panel). Taken together the data show that the site required for RNase II–RNaseE association is present within the RNaseE**^(696–1021)^** fragment that includes the RhlB-binding site. This is consistent with the observation that RNase II–RNaseE Co-IP required expression of both RhlB and the complete RNaseE^C^ scaffold domain which also includes the RhlB-binding site (Figures 2 and 4). Attempts to further narrow the RNaseE fragment that mediates the RNase II–degradosome association were not successful because of inability to stably express other shorter RNaseE^C^ fragments.

The failure of RNase II to interact with RNaseE^C^ fragments that lack the RhlB-binding site appeared not to be due to global misfolding or instability of the fragments as indicated by the observation that the RNaseE^C^ fragments maintained their ability to interact with other degradosomal proteins. This was shown by anti-HA IP experiments on extracts containing plasmid-encoded RNaseE^C^ fragments that lacked the RhlB-binding site (RNaseE**^(820–1061)^**–HA) (Figure 5E) or that lacked both the RhlB- and PNPase-binding sites (RNaseE**^(762–1021)^**–HA) (Figure 5G). In both cases, the expected PNPase and Eno bands were present in the IP fractions (Figure 5E, G left panels), whereas the RNase II-Flag band was absent (Figures 5E and 5G right panels). This indicates that the RNaseE fragments that failed to co-immunoprecipitate with RNase II were fully functional in their ability to interact with other protein partners, and presumably had not undergone global misfolding or degradation.

### Role of the RNaseE membrane-binding domain in the RNaseE–RNase II association

**Figure 6:**
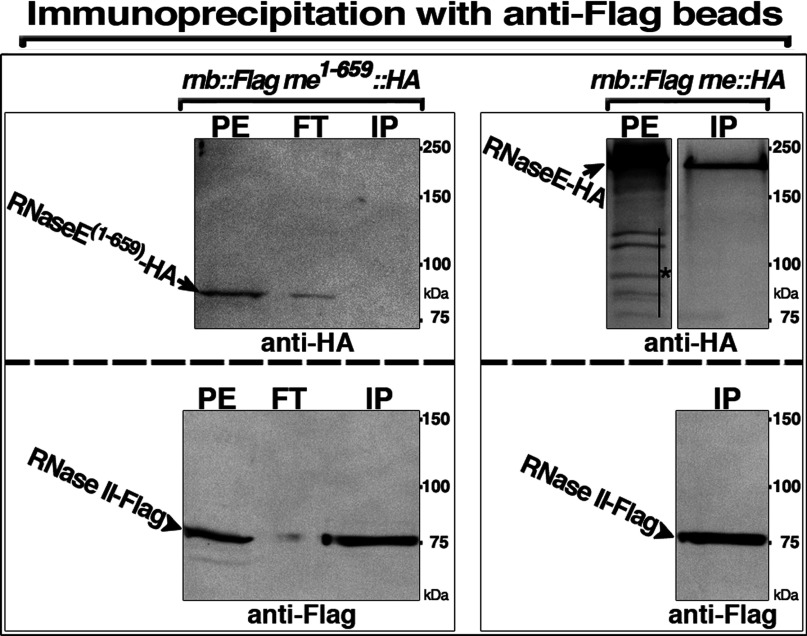
Role of the RNaseE membrane-binding domain in the RNaseE–RNase II association IP with anti-Flag beads of PEs from cells that coexpressed under control of the native promoters chromosomally encoded RNase II–Flag and RNaseE**^(1–659)^**–HA (left panels) or RNase II–Flag and RNaseE–HA (right panels). In these cells, genes encoding the indicated epitope-tagged proteins replaced the corresponding wild-type genes. PE and the obtained IP fractions, flow-through (FT) and bound (IP), were subjected to Western blot using anti-HA (top panels) or anti-Flag (bottom panels) polyclonal antibodies. Arrows indicate positions of bands corresponding to RNaseE–HA or RNaseE**^(1–659)^**–HA; arrowheads indicate positions of the RNase II–Flag bands; and star indicates bands that correspond to RNaseE–HA breakdown fragments. The shown panels are from one gel. Protein size is shown in kDa.

RNase II and RNaseE are membrane-associated proteins whose binding to the cytoplasmic membrane requires an intrinsic amphipathic helix [23,34]. This raised the possibility that the Co-IP of RNase II and RNaseE might have come from non-specific independent binding of the two proteins to residual small membrane or lipid vesicles or to lipid-detergent micelles formed during preparation of the PEs. This possibility was excluded by the demonstration that RNaseE**^(1–659)^**, which lacks most of the RNaseE^C^ fragment but retains the membrane-anchor (A motif, Figure 5A) required for membrane binding [29,34] failed to associate with RNase II in Co-IP experiments (Figure 6). Moreover, several RNaseE fragments that lacked the RNaseE membrane-anchor but retained the RhlB-binding site (e.g., RNaseE**^(634–1021)^**–HA) retained the RNaseE–RNase II association in Co-IP experiments (Figures 5A and 5C). These experiments appear to exclude the role of membrane in bridging RNaseE and RNase II in the Co-IP and far Western experiments.

Maintenance of interaction of RNase II with the RNaseE that lack the membrane-binding domain does not exclude a role of membrane-association of the proteins in the RNase II–RNaseE interaction. Lipid-detergent or detergent micelles present in the reaction mixtures could presumably substitute for the membrane that could play a role in increasing the local concentration or inducing changes in conformation of the associated proteins. The role of membrane binding in the RNase II–degradosome association remains to be established.

## DISCUSSION

The present work shows that RNase II, the major hydrolytic exoribonuclease of *E. coli*, is associated with the RNA degradosome. The RNase II–degradosome association was shown by Co-IP of RNase II with all of the canonical degradosome proteins and by gel affinity blots using purified RNaseE–HA as bait and cell extracts containing RNase II–Flag as prey. RNaseE–HA and RNase II–Flag were functional in complementation assays of *∆rne* or *∆*(*rnb pnp*) cells, respectively [23,29]. The RNase II–degradosome association is not mediated by binding of the proteins to a common RNA substrate as shown by its insensitivity to RNase pretreatment. Further evidence that RNase II is associated with the degradosome came from the finding that other degradosome components, primarily RhlB, are required for interaction of RNase II with the core degradosomal protein RNaseE. The association of RNase II with the degradosome complex is consistent with the colocalization of RNase II and RNaseE within higher-order organized cellular structures visualized by fluorescence microscopy ([35] and reviewed in [36]). We conclude that RNase II is a component of the RNA degradosome.

Loss of degradosome assembly was associated with global changes in normal mRNA turnover [2] and protein synthesis [1] in cells that lack the RNaseE C-terminal scaffold domain, RhlB, or PNPase, which are required for formation of the RNase II–degradosome complex described here. RNase II, a hydrolytic exoribonuclease, accounts for 90% of exoribonuclease activity of cell extracts, suggesting that *E. coli* RNA degradation is a predominantly hydrolytic [6]. The addition of RNase II to the panoply of degradosome proteins may explain the paradox that RNA degradation in *E. coli* cell extracts is primarily hydrolytic [6] in face of the previous assumption the degradosome that is required for maintenance of the normal half-life of cellular transcripts [2,37] contained only a phosphorolytic exoribonuclease, PNPase.

What might be the role of the RNase II–degradosome association? RNase II and PNPase play crucial and overlapping roles in normal cell physiology as shown by the observation that cells can survive in the absence of one or the other of these enzymes, whereas cells that lack both enzymes are not viable [12]. However, it is not known how or whether the exoribonuclease activities of RNase II and the degradosome-associated PNPase are coordinated. The association of PNPase and RNase II within a single RNase II–degradosome complex could provide a mechanism to coordinate the RNase II and PNPase activities within the RNA degradation pathway. This might be achieved, for example, if the RNA substrates processed by the RNaseE and/or RhlB elements of the complex were selectively presented to either PNPase or RNase II depending on the specific RNA [38–40] or other factors.

RNase II may be associated with the degradosome by direct binding to RNaseE, which also contains binding sites for the other degradosome proteins. Alternative possibilities include tripartite interactions between RNase II and other degradosome components that could act as linkers in attachment of RNase II to RNaseE, or conformational changes in RNaseE upon binding RhlB and PNPase that structure or expose a binding site for RNase II. Although neither possibility can be definitively excluded, the data seem to favour the direct binding of RNase II to RNaseE. Thus, although PNPase alone was capable of partially supporting the RNase II–RNaseE association (Figure 4C panel 3, 4F lane 2), PNPase failed to support the association of RNase II with an RNaseE fragment (RNaseE**^(820–1061)^**) that contained the binding site for PNPase but not for RhlB (Figure 5E). This showed that the binding of PNPase to RNaseE was not sufficient to attach RNase II to the degradosome.

**Figure 7:**
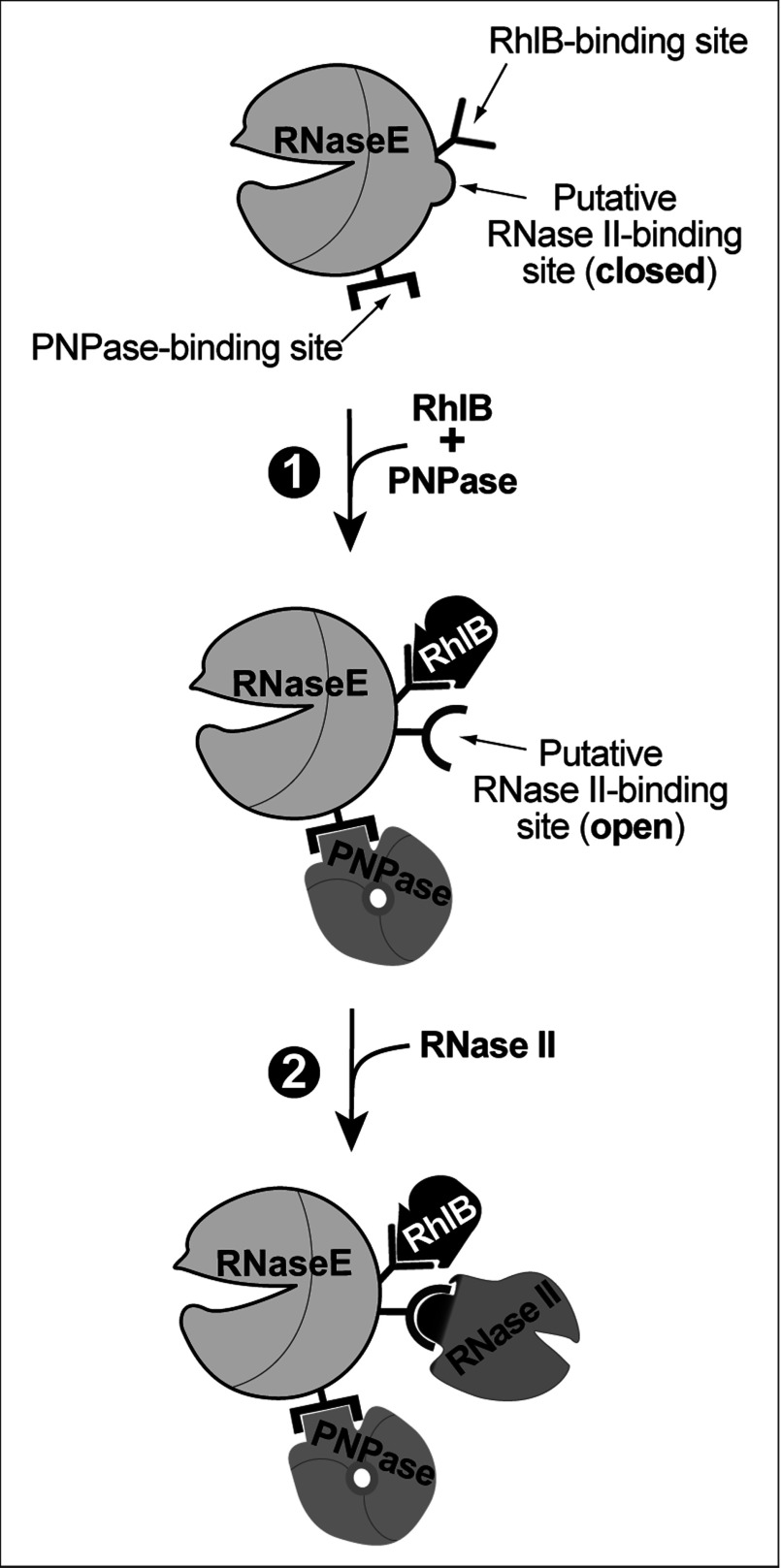
Proposed model for assembly of the RNase II–degradosome complex RNaseE is known to contain binding sites for RhlB (V-shape), PNPase (bracket-shape) and Eno (not shown for simplicity). RhlB and PNPase bind to their sites and induce a conformational change that converts the RNase II-binding site from a closed to an open conformation. RNase II binds to the exposed RNase II-binding site.

The present results seem most compatible with a model in which an RNase II-binding site is present in the RNaseE**^(696–762)^** fragment which also contains the RhlB-binding site (Figure 7). Accessibility of RNase II to its binding site would be dependent on a conformational change induced by RhlB binding and, perhaps to a lesser extent, by PNPase binding to the RNaseE scaffold domain. Further work will be required to establish the molecular basis of the RNase II–degradosome association.
